# Absence of TFE3 Immunoexpression in a Spectrum of Cutaneous Mixed Tumors: A Retrospective Pilot Study

**DOI:** 10.3390/dermatopathology9010008

**Published:** 2022-01-29

**Authors:** Hatice B. Zengin, Bahadir Yildiz, Tatsiana Pukhalskaya, Bruce R. Smoller

**Affiliations:** Department of Pathology and Laboratory Medicine, University of Rochester Medical Center (URMC), Rochester, NY 14642, USA; Bahadir_Yildiz@URMC.Rochester.edu (B.Y.); Tatsiana_Pukhalskaya@URMC.Rochester.edu (T.P.); Bruce_Smoller@URMC.Rochester.edu (B.R.S.)

**Keywords:** chondroid syringoma, malignant chondroid syringoma, cutaneous mixed tumor, TFE3

## Abstract

Background: Cutaneous mixed tumors (CMTs) include benign, atypical, and malignant chondroid syringomas. This spectrum of entities is known to be a part of myoepithelial neoplasms, which display considerable genetic heterogeneity. In a previous report, a malignant chondroid syringoma (MCS) demonstrated PHF1-TFE3 gene fusion and strong TFE3 immunohistochemical (IHC) staining. The authors suggested that the MCS is genetically related to tumors with TFE3 rearrangements such as renal cell carcinoma and might have genetic heterogeneity. In this study, we aim to investigate potential TFE3 gene fusions with TFE3 IHC stain in a spectrum of CMTs. Materials: Eleven benign chondroid syringoma (BCS), one atypical chondroid syringoma (ACS), and one malignant chondroid syringoma cases were identified, stained with TFE3 IHC stain, and interpreted based on preset criteria. Results: ACS and MCS cases did not show any staining. In 7 of 11 BCS cases, weak (1+) staining was observed in less than 20% of the tumor cells and were considered negative. Additionally, in one BCS case, weak (1+) and (2+) staining was shown in approximately 15% and less than 1% of the tumor cells, respectively. Based on our positivity criteria, this case was also interpreted as negative. Conclusions: Our study failed to reveal possible TFE3 gene fusion by IHC staining in benign, atypical, and malignant chondroid syringomas. Although the negative staining in MCS suggests a genetic heterogeneity in this entity, further studies with larger case groups are needed for a more definitive conclusion.

## 1. Introduction

Transcription factor enhancer 3 (TFE3) gene is located on the short arm of chromosome Xp11.23 and belongs to microphthalmia transcription family (MiTF) [1]. It encodes a basic helix-loop-helix domain-containing transcription factor that binds MUE3-type E-box sequences in the promoter of genes and operates transcription [1]. TFE3 gene fusions with variable gene partners occur in many neoplasms including renal cell carcinoma and lead to immunohistochemical (IHC) nuclear staining [1]. Recently, a new gene fusion of plant homeodomain (PHD) finger protein 1 gene (PHF1) to TFE3 was identified in a malignant chondroid syringoma [2].

Chondroid syringomas (CSs), also called cutaneous mixed tumors, are rare neoplasms composed of epithelial/myoepithelial cells distributed into a myxoid, chondroid, and fibrous stroma [3]. Benign CS is known to be a well-circumscribed tumor, as is an atypical mixed tumor, which may additionally show some architectural or cytological atypia without capsular invasion [3]. Less than 50 malignant CS cases have been published in the literature where they develop either from benign CS or de novo [3]. The epithelial component of this malignant entity may exhibit variable pleomorphism and scattered mitoses [3]. Not long ago, pleomorphic adenoma gene 1 (PLAG1) rearrangements were the only genetic alterations found in chondroid syringomas [4,5]. The PHF1-TFE3 gene fusion in malignant CS suggested a genetic heterogeneity in this group. It also advocated a relationship between malignant CS and the other TFE3 rearranged tumors, and necessitated further investigation. We hypothesize that TFE3 gene alterations might be common in this disease and may have prognostic as well as diagnostic significance. Our goal in this study is to analyze a spectrum of CSs for TFE3 IHC nuclear staining and enhance our knowledge about genetic alterations in these entities.

## 2. Materials and Methods

This Institutional Review Board (IRB)-approved study was based on the retrospective analysis of the archival tissue. Surgical pathology specimens diagnosed with benign chondroid syringoma (CS), atypical chondroid syringoma, and malignant chondroid syringoma between the dates of 1 January 2013 and 1 October 2021 were reviewed by the authors for inclusion. Inclusion criteria included biopsies from adult patients with diagnostic histologic findings. However, only one case of atypical chondroid syringoma and one case of malignant chondroid syringoma were identified. Eleven cases that met the diagnostic criteria for benign chondroid syringoma were randomly selected to be included the study. A total of 13 biopsies from benign CS (11), atypical CS (1), and malignant CS (1) were collected. Additionally, biopsy site, patients’ age and gender, as well as follow-up information, were noted (Table 1).

Immunohistochemical (IHC) studies were performed on 4 µm sections of formalin-fixed, paraffin-embedded tissue using the Leica Bond III instrument IHC assay consisted of TFE3 Leica clone MRQ-37 (Cell Marque, Rocklin, CA, USA) in a dilution of 1:100. After incubation for 60 min, the epitope retrieval was performed using high pH. Tissue staining was performed on a Leica BOND III immunostainer with a Leica Refine Polymer Detection Kit. A section from a translocation-associated renal cell carcinoma was used as an external positive control.

The positivity of TFE3 was defined as dark-brown nuclear staining. The density of the staining was graded as weak (1+), moderate (2+), and strong (3+). Cases were interpreted as strongly positive if there was any percentage of strong staining (3+), weakly positive if there was weak (1+) staining in more than 20% of tumor cells, or if there was moderate staining (2+) in more than 5% of the tumor cells. Cases were addressed as negative if there was no staining, weak (1+) staining in less than 20% of tumor cells, or moderate (2+) staining in less than 5% of tumor cells. Positive cases were further subcategorized into patchy or diffuse staining if the positive neoplastic cells comprised <50% or >50% of the entire tumor cell count, respectively.

## 3. Results

**Figure 1:**
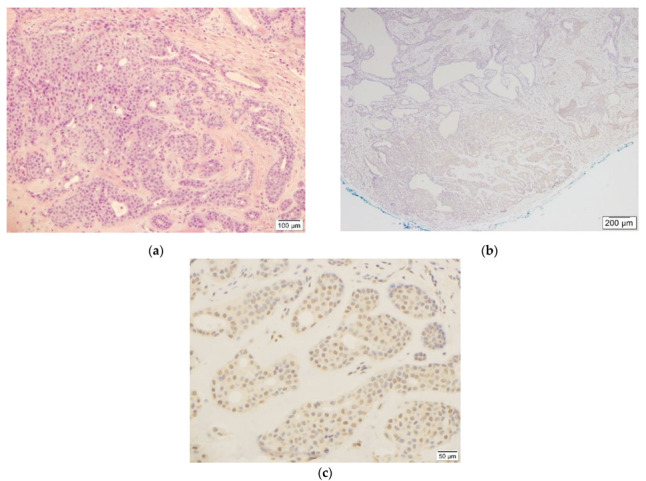
This figure shows benign chondroid syringoma ((**a**)**,** H&E, 10×, case #1) and weak (1+) TFE3 staining ((**c**), TFE3, 20×) in less than 20% of tumor cells in the corresponding area ((**b**), TFE3, 4×).

In the benign CS group, seven out of eleven cases showed weak (1+) staining in less than 20% of the neoplastic cells (Figure 1 and Figures S1–S6) and were considered negative results.

**Figure 2:**
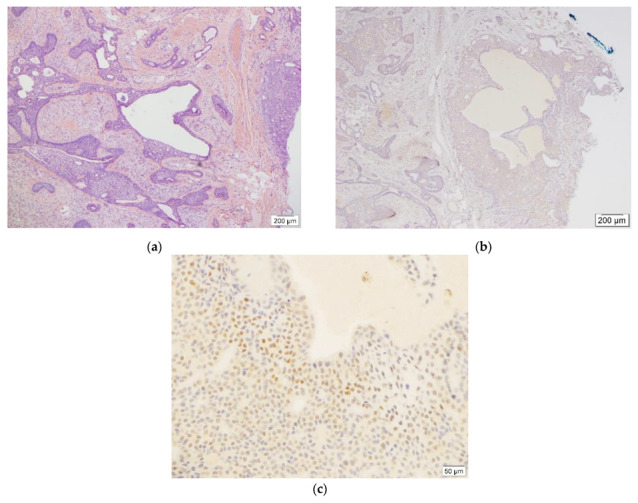
This figure demonstrates benign chondroid syringoma ((**a),** H&E, 4×, case #2) and weak (1+) as well as adjacent moderate (2+) TFE3 staining ((**c**), TFE3, 20×) in less than 20% and 5% of tumor cells, respectively ((**b**), TFE3, 4×).

One case demonstrated weak (1+) staining in 15% and moderate (2+) staining in 1% of the entire tumor cells (Figure 2). The remaining three cases were completely negative for TFE3 IHC stain (Figure S7–S9). In this group, none of the cases met the positivity criteria and were reported as negative.

The study included only one (1) atypical CS case. The neoplastic cells in this biopsy did not reveal any staining for TFE3 IHC stain (Figure S10). The case was categorized as negative.

**Figure 3:**
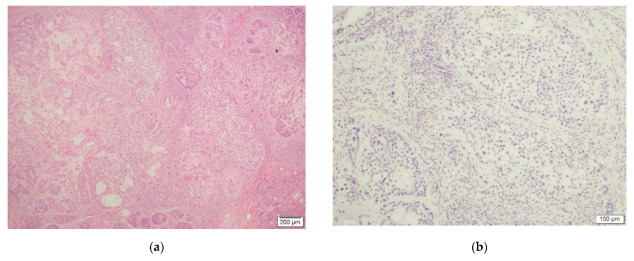
This figure depicts malignant chondroid syringoma ((**a**), H&E, 4×, case #13) and negative TFE3 IHC staining ((**b**), TFE3, 10×).

Lastly, in one (1) malignant CS case, the tumor cells were entirely negative for the stain and the case was reported as negative as well (Figure 3).

## 4. Discussion

In this study, we used TFE3 IHC stain to detect possible gene fusions in chondroid syringomas (CSs). In contrast to Panagopoulos’ et al. [2] report, the tumor cells in our malignant CS case did not reveal any staining with TFE3. Our study showed that the TFE3 gene fusions may not occur in a portion malignant CSs and further supported the genetic heterogeneity in this entity. We also analyzed the other members of this group of tumors with such differentiation, atypical and benign chondroid syringomas, with the same IHC stain. Although we detected foci of weak staining in some benign CSs, none of the cases met our positivity criteria. Considering the relatively large group of benign cases in our study, it is possible that TFE3 gene rearrangements might be specific, yet not highly sensitive to malignant CSs. On the other hand, we were not able to corroborate the findings of the previous study [2] with our single malignant example. However, it is still possible that a positive TFE3 stain could be helpful in differentiating the malignant cases from benign counterparts. Our limited case number should be noted and additional studies are needed to support this thesis.

Chondroid syringomas are considered a cutaneous part of myoepithelial neoplasms that show a variety of genetic alterations, such as gene fusion of EWSR1 with PBX1 in soft tissue originated tumors [6,7]. In cutaneous myoepithelial neoplasms, PLAG1 gene rearrangements in addition to its fusions with N-myc downstream regulated 1 (NDRG1) and transcriptional repressor GATA binding 1 (TRPS1) genes have been demonstrated [6,8]. Panagopoulos et al. reported the first nuclear TFE3 IHC staining as well as PHF1-TFE3 gene fusion in malignant CS [2]. Based on this finding, the authors postulated that there might be a genetic link between malignant chondroid syringomas and PHF1 or TFE3 altered tumors. They also emphasized the necessity of analyzing more CS cases for the above-mentioned and probable many more genetic abnormalities. 

PHF1 gene plays a role in regulating gene transcription and is involved in variable neoplasms with its fusion partners [2,9]. It was first reported as JAZF1-PHF1 and EPC1-PHF1 gene fusions in low-grade endometrial stromal sarcomas [2]. Later, MEAF6-PHF1, BRD8-PHF1, and EPC2-PHF1 fusions have also been demonstrated [2]. Additionally, PHF1 gene rearrangements and EP400-PHF1, EPC1-PHF1, and MEAF6-PHF1 fusion genes have been shown in ossifying fibromyxoid tumors [9]. 

Likewise, TFE3 gene fusions also account for important genetic alterations in diverse tumors. After their discovery in papillary renal cell carcinoma (PRCC) as PRCC-TFE3 gene fusion, further partnerships have been reported, including NONO, SFPQ, and DVL2 genes in papillary renal cell carcinomas and PEComas [2]. Moreover, ASPSCR1-TFE3 in alveolar soft part sarcomas and papillary renal cell carcinomas, and YAP1-TFE3 in epithelioid hemangioendotheliomas have been demonstrated [2]. Immunohistochemical stains can highlight the nuclear TFE3 protein in tumors with or without TFE3 gene alterations [1]. Granular cell tumor and solid pseudopapillary neoplasm of pancreas also express TFE3 stain with no associated genetic abnormalities [1]. Nevertheless, the nuclear TFE3 staining malignant CS represents an underlying gene fusion [2].

Although PLAG1 alterations in CSs and PHF1-TFE3 gene fusion in malignant counterparts have been described, our knowledge about genetic abnormalities in these tumors is limited. However, it is sensible that a broad genetic variability exists in CSs as it does in other myoepithelial neoplasms. Our results suggests that at least a portion of benign cases and one malignant case do not show TFE3 gene rearrangements. We believe that it may be limited to malignant CSs with an indeterminate sensitivity. Further studies are needed to clarify the genetic profile in this entity. Hence, it may help in diagnosis but more importantly, in determining variable clinical presentations with other neoplasms and course of disease.

## Figures and Tables

**Table 1:** Patients’ demographic and follow-up information.

Category	Case Number	Age	Gender	Biopsy Site	Treatment/Follow-Up
Benign	1	66	Male	Scalp	Complete excision/No recurrence for 51 months
	2	25	Female	Upper lip	Complete excision/No recurrence for 54 months
	3	32	Male	Nasal area	Complete excision/Recurrence on 15th month
	4	74	Female	Right lateral eyebrow	Complete excision/Not available
	5	65	Male	Right upper forehead	Complete excision/No recurrence for 43 months
	6	44	Male	Left chin	Complete excision/No recurrence for 12 months
	7	52	Female	Left nasal ala	Complete excision/Not available
	8	38	Male	Right nasal skin area	Complete excision/No recurrence for 11 months
	9	39	Female	Right lateral shin	Complete excision/No recurrence for 6 months
	10	34	Female	Right parietal scalp	Complete excision/No recurrence for 6 months
	11	51	Male	Forehead	Complete excision/No recurrence for 34 months
Atypical	12	61	Male	Right cheek	Complete excision/No recurrence for 11 months
Malignant	13	82	Female	Right dorsal hand	Complete excision/No recurrence for 3 months

## Data Availability

All data generated or analyzed during this study are included in this article and its supplementary material files. Further enquiries can be directed to the corresponding author.

## Supplementary Materials

The following supporting information can be downloaded at: https://www.mdpi.com/article/10.3390/dermatopathology9010008/s1, Figure S1: This figure shows weak (1+) TFE3 staining in less than 20% of tumor cells in case #5 with benign features (a—10×, b—20×). Figure S2: This figure shows weak (1+) TFE3 staining in less than 20% of tumor cells in case #6 with benign features (a—4×, b—20×). Figure S3: This figure shows weak (1+) TFE3 staining in less than 20% of tumor cells in case #7 with benign features (a—4×, b—20×). Figure S4: This figures shows weak (1+) TFE3 staining in less than 20% of tumor cells in case #8 with benign features (a—10×, b—20×). Figure S5: This figure shows weak (1+) TFE3 staining in less than 20% of tumor cells in case #9 with benign features (a—10×, b—20×). Figure S6: This figure shows weak (1+) TFE3 staining in less than 20% of tumor cells in case #10 with benign features (a—10×, b—20×). Figure S7: This figure shows negative TFE3 staining in case #3 with benign features (a—10×, b—20×). Figure S8: This figure shows negative TFE3 staining in case #4 with benign features (a—10×, b—20×). Figure S9: This figure shows negative TFE3 staining in case #11 with benign features (a—10×, b—20×). Figure S10: This figure shows negative TFE3 staining in case #12 with atypical features (a—10×, b—20×).
