# Supplementary material for: Absence of TFE3 Immunoexpression in a Spectrum of Cutaneous Mixed Tumors: A Retrospective Pilot Study
Source: Dermatopathology (Basel). 2022 Jan 29;9(1):48–53. doi: 10.3390/dermatopathology9010008 (PMC8883959; doi:10.3390/dermatopathology9010008)
Supplement: Supplementary file 1 [file dermatopathology-09-00008-s001.zip › dermatopathology-1481307-supplementary.pdf]

dermatopathology

Supplementary Figures

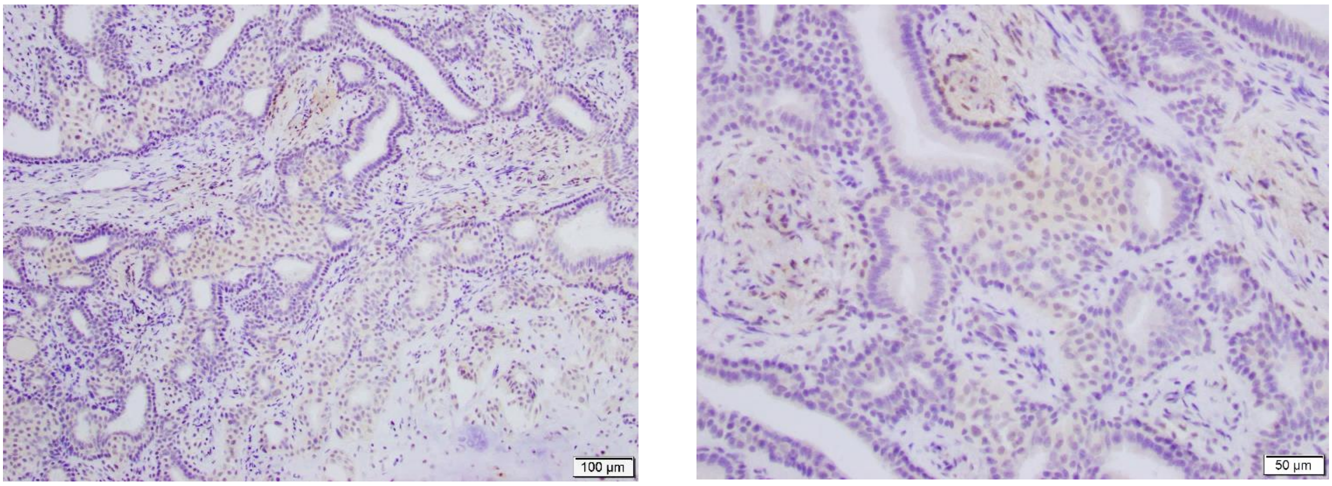

(**a**) (**b**)

Figure S1. This figure shows weak (1+) TFE3 staining in less than 20% of tumor cells in case #5 with benign features (a - 10X, b - 20X).

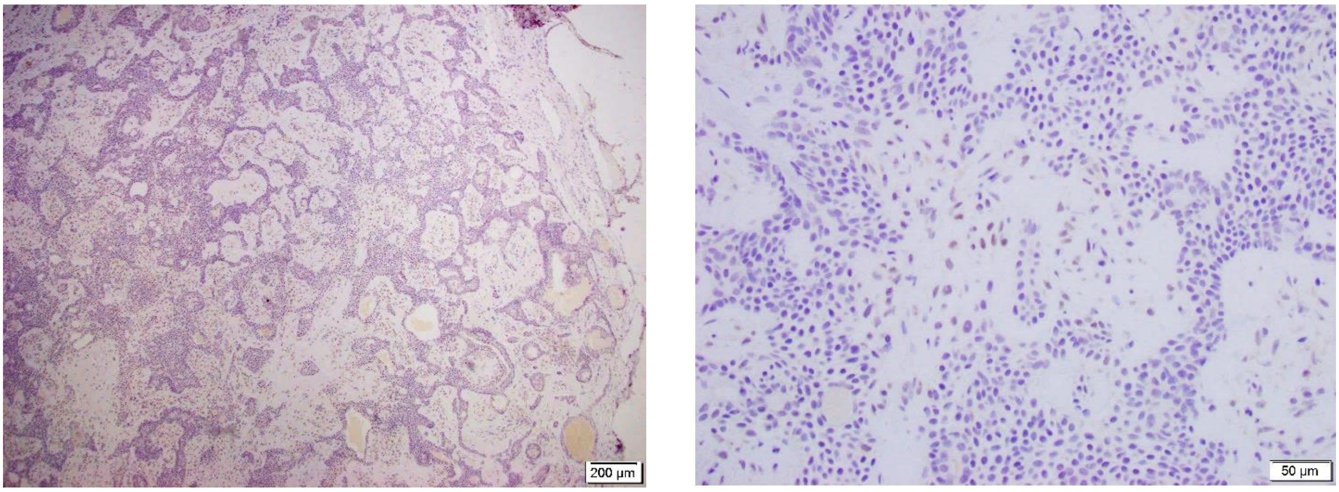

(**a**) (**b**)

Figure S2. This figure shows weak (1+) TFE3 staining in less than 20% of tumor cells in case #6 with benign features (a - 4X, b - 20X).

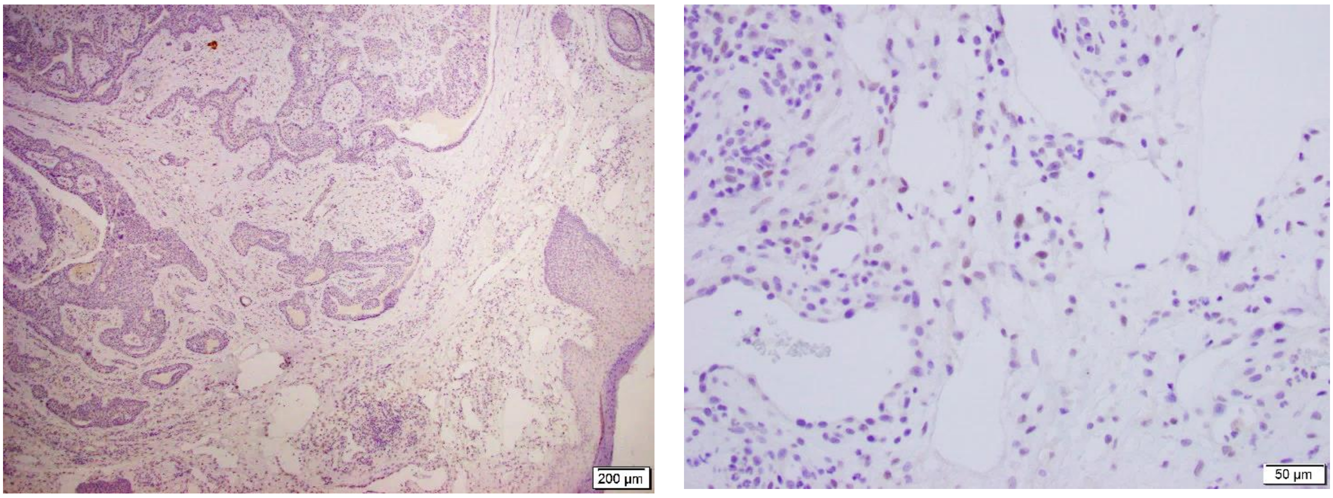

(**a**) (**b**)

Figure S3. This figure shows weak (1+) TFE3 staining in less than 20% of tumor cells in case #7 with benign features (a - 4X, b - 20X).

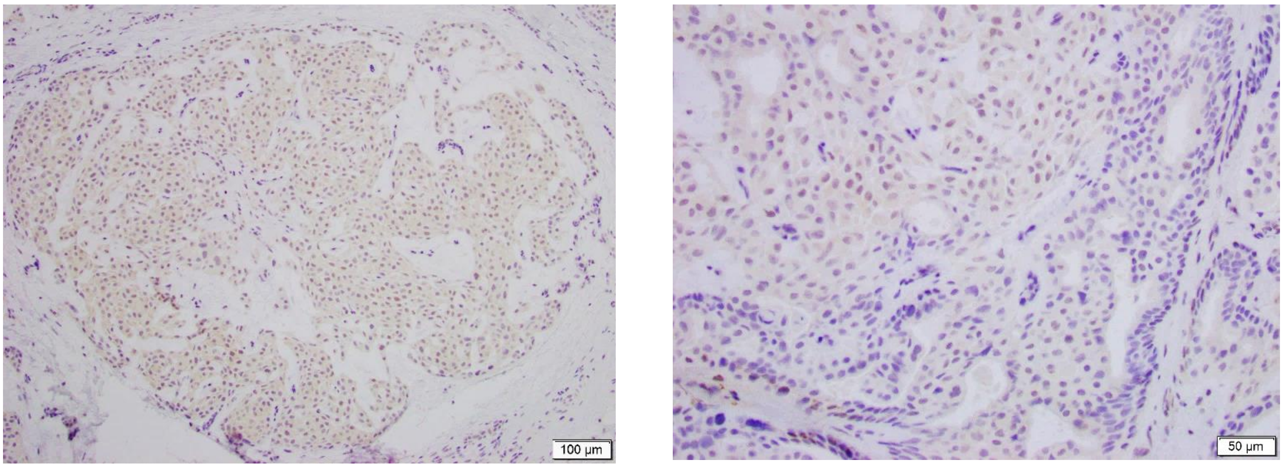

(**a**) (**b**)

Figure S4. This figures shows weak (1+) TFE3 staining in less than 20% of tumor cells in case #8 with benign features (a - 10X, b - 20X).

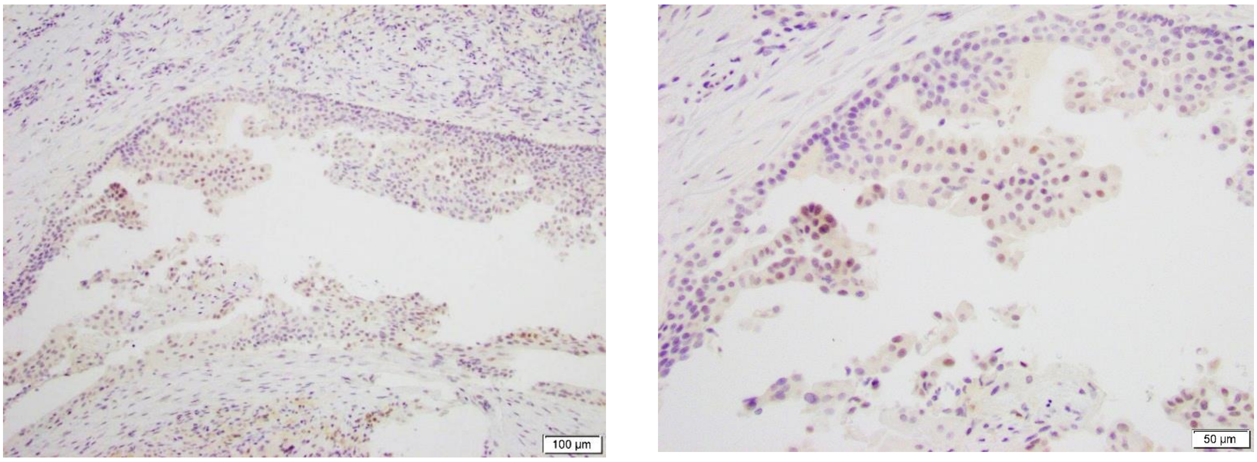

(**a**) (**b**)

Figure S5. This figure shows weak (1+) TFE3 staining in less than 20% of tumor cells in case #9 with benign features (a - 10X, b - 20X).

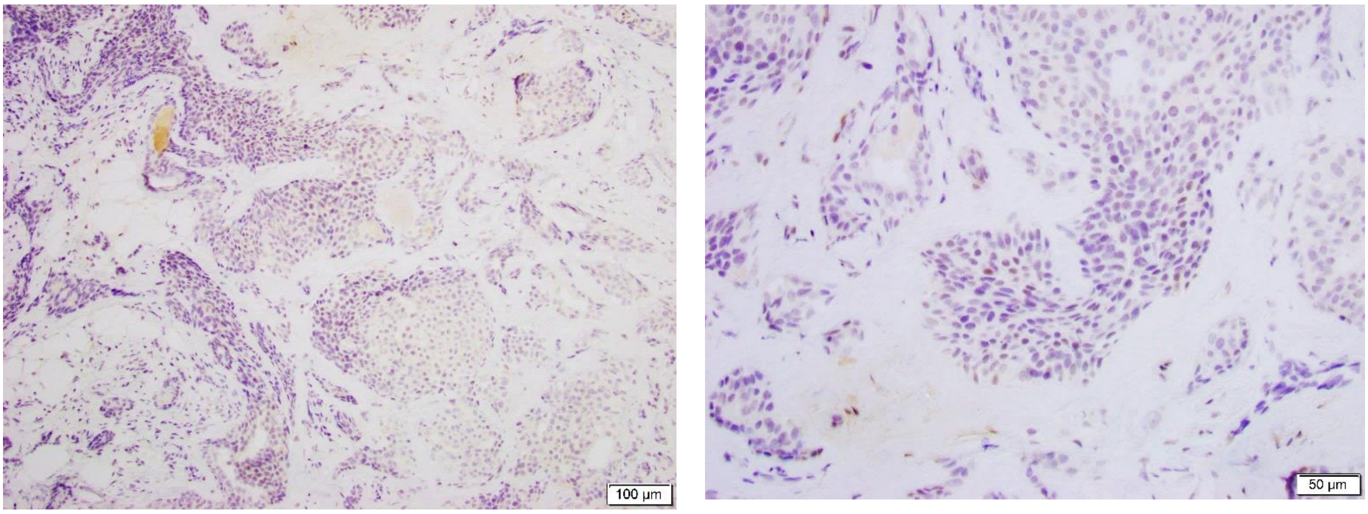

(**a**) (**b**)

Figure S6. This figure shows weak (1+) TFE3 staining in less than 20% of tumor cells in case #10 with benign features (a - 10X, b - 20X).

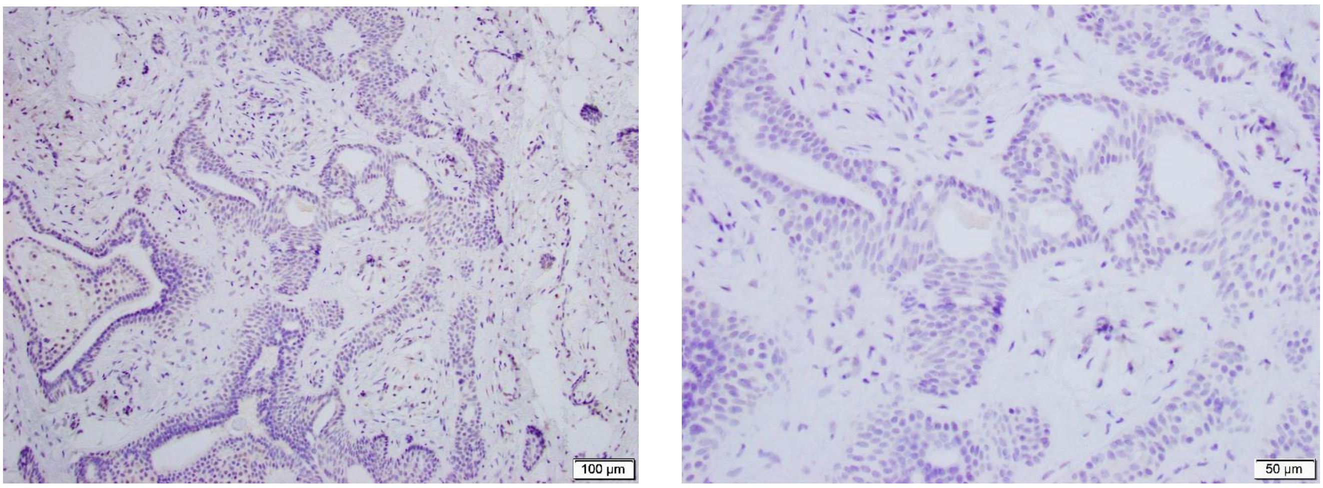

(**a**) (**b**)

Figure S7. This figure shows negative TFE3 staining in case #3 with benign features (a - 10X, b - 20X).

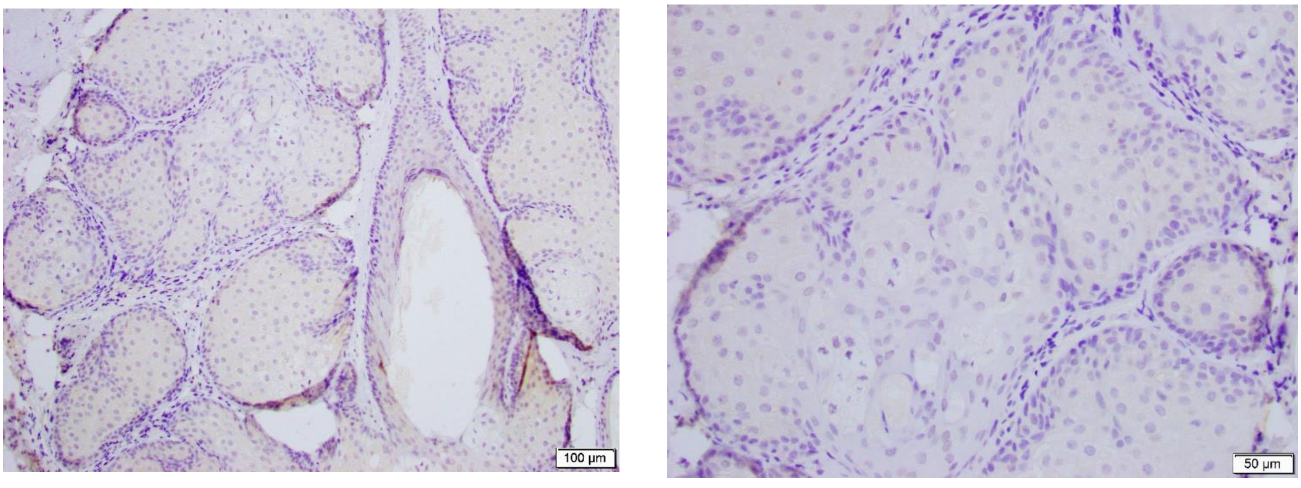

(**a**) (**b**)

Figure S8. This figure shows negative TFE3 staining in case #4 with benign features (a - 10X, b - 20X).

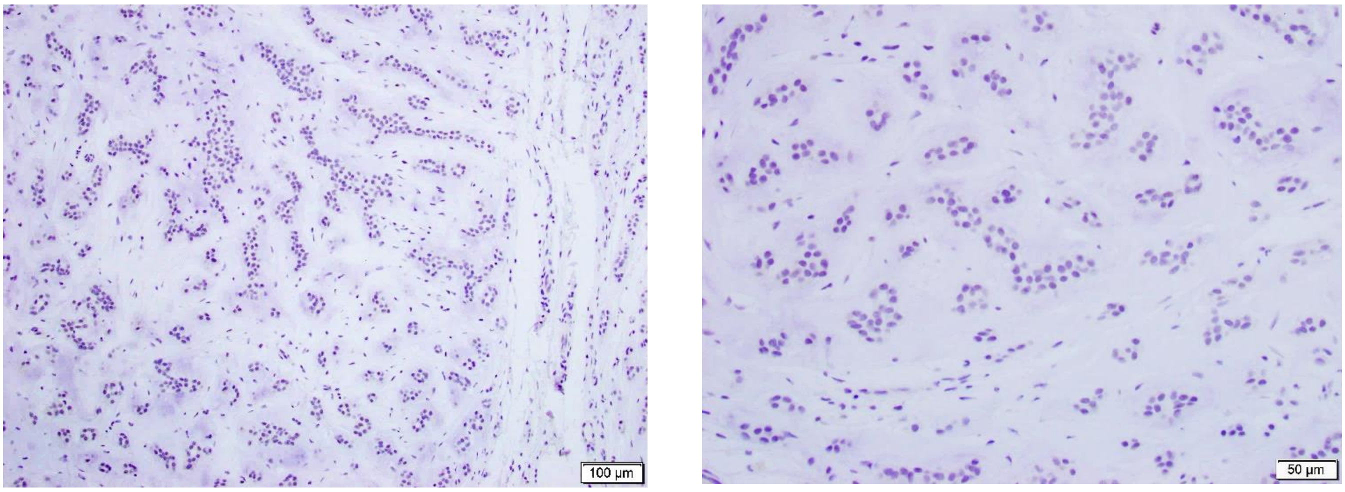

(**a**) (**b**)

Figure S9. This figure shows negative TFE3 staining in case #11 with benign features (a - 10X, b - 20X).

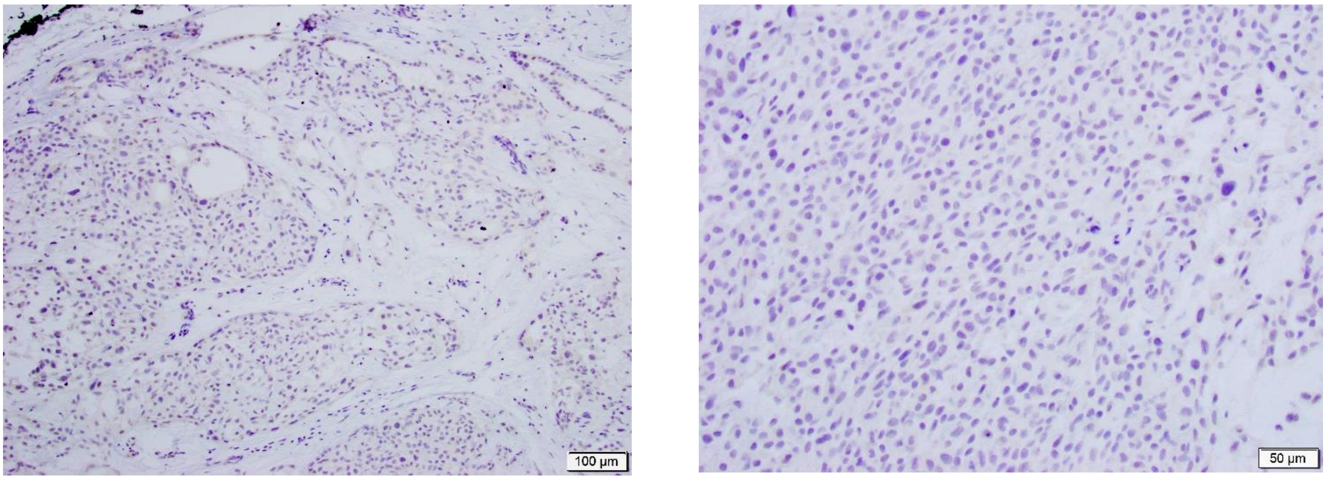

(**a**) (**b**)

Figure S10. This figure shows negative TFE3 staining in case #12 with atypical features (a - 10X, b - 20X).
